# Pain and the autonomic nervous system. The role of non-invasive neuromodulation with NESA microcurrents

**DOI:** 10.3389/fpain.2025.1410808

**Published:** 2025-02-17

**Authors:** Nelson Azevedo, Raquel Medina-Ramírez

**Affiliations:** ^1^ ISAVE, Amares, Portugal; ^2^ CIR, ESS, Polytechnic of Porto, Porto, Portugal; ^3^ SocDig Research Group, University of Las Palmas de Gran Canaria, Las Palmas, Spain

**Keywords:** pain, autonomic nervous system, neuromodulation, NESA, microcurrents

## 1 Introduction

Human pain is a complex and multifaceted phenomenon (1) that is controlled by multiple systems in the human body (2). These systems work together to process, interpret and respond to pain (3).

The International Association for the Study of Pain defines pain as “an unpleasant sensory and emotional experience associated with, or resembling that associated with, actual or potential tissue damage” (4). This experience evokes an integrated response from the various systems associated with pain.

This integrated response to the phenomenon of pain occurs in a more complex way at the level of the cortex where pain is consciously perceived, such as the primary somatosensory cortex, the primary motor cortex and supplementary motor cortex, the secondary somatosensory cortex, the insular cortex, the anterior cingulate cortex and the thalamus (5, 6). Also at an unconscious level, but more central, is the emotional regulation of pain, which is linked to the limbic system, where the experience of pain acquires an individual component linked to the emotions and experiences of the individual themselves, such as memory and fear in the amygdala, hippocampus and subcortical structures, including the basal ganglia (6, 7).

In addition, there are some systems that are particularly important in pain regulation, namely the immunological system, the endocrine system and the descending noradrenergic system.

Despite today's deeper understanding of pain mechanisms, there is still a lack of solutions for more complex conditions. The role of the autonomic nervous system (ANS) in pain modulation with the different systems involved in pain, such as the immunological system, the endocrine system and the descending noradrenergic system, is becoming increasingly clear and there is an urgent need to develop solutions for effective modulation of the ANS with a focus on pain (9). From this perspective, neuromodulation of the ANS appears to be a potentially supportive solution for the modulation of pain, in particular non-invasive neuromodulation of the autonomic nervous system NESA, which has been shown to be effective for pain in chronic pathologies such as multiple sclerosis (8).

The need arises for effective non-pharmacological treatments, based on electrical neuromodulation, and backed by scientific evidence to support their use for pain. In this sense, previous studies have shown that microcurrent technologies for prefrontal and primary motor cortex modulation such as transcranial stimulation (tDCS) combined with peripheral stimulation (9) or vagal stimulators (VNS) (10) have demonstrated benefits on different aspects of pain, headaches, chronic pain in diverse populations (11). However, science is advancing and other types of specific microcurrent are emerging for autonomic modulation targets that are more comfortable for the patient, with no side effects and an easier application, such as non-invasive neuromodulation NESA (12). It is therefore necessary to explore the field of work of NESA microcurrents so that they can be used to modulate pain through the autonomic nervous system.

## 2 Subsections relevant for the subject

The immune system influences the release of substances such as pro-inflammatory cytokines, which sensitize pain receptors and pathways and thus increase the perception of pain (13). The immune system also plays a role in reducing pain levels in the process of tissue recovery (14). The regulation of the immune system, pain and inflammation is largely mediated by the ANS and its different divisions such as the sympathetic, parasympathetic and enteric branches (15).

This deregulation can be observed in autoimmune diseases such as rheumatoid arthritis, systemic lupus erythematosus and multiple sclerosis, where changes in the ANS are associated with increased inflammation. As a result of these increased inflammatory levels, there is a dysfunctional interaction between the ANS and the gut microbiota with direct effects on the homeostasis of inflammation (14, 15). A study with individuals with rheumatoid arthritis showed a strong correlation between the increase in sympathetic activity due to muscle sympathetic nerve activity and the painful symptoms of these patients (16).

The endocrine system also has an influence on pain regulation via hormones that influence pain perception (17, 18). The hypothalamic-pituitary-adrenal axis (HPA axis) and cortisol is an important hormonal regulatory system that is also of great importance for the regulation of pain by regulating cortisol levels. Elevated cortisol levels following acute stress can facilitate the consolidation of fear-based emotional memories and cause a sensitized physiological response to stress. However, prolonged stress or situations of constant or exaggerated physical pain can increase sympathetic and neuroendocrine activity, decrease cortisol levels, and perpetuate generalized pain and inflammation (19). The increase in cortisol levels is accompanied by an increase in adrenaline levels associated with acute pain or stress, which can transiently alter pain perception (14). This type of response is often associated with the “fight or flight” response, which can temporarily reduce pain sensitivity (20). However, prolonged chronic stress leads to a functional change in the ANS that impairs pain regulation, such as in fibromyalgia (21, 22) and central sensitization (23).

Pain modulation also depends on an important system such as the descending noradrenergic system, its relationship with the periaqueductal gray matter (PAG) and the reticular formation of the brainstem (24). The PAG is also involved in risk assessment and threat response and contributes to defensive behaviors related to negative events. These pathways lead to learned aversive behaviors through the involvement of sympathetic responses, motor responses, and emotional responses characteristic of acute and chronic pain (25). Some structures are particularly important for the development and maintenance of allodynia and hyperalgesia in nerve tissue damage, such as the noradrenergic neurons in the locus coeruleus and their terminals in the dorsal reticular nucleus, medial prefrontal cortex, dorsal horn of the spinal cord and spinal caudal trigeminal nucleus (26).

The brainstem, in particular the various nuclei of the reticular formation such as the locus coeruleus and the serotonergic nuclei of the raphe, play a fundamental role in the modulation of pain, but also in the regulation of sleep together with the ANS. Wakefulness and sleep are behaviors characterized by different levels of engagement with the external environment. Accordingly, there is a consistent remodulation of ANS activity during the transition from wakefulness to sleep (27). Recent qualitative analyzes of longitudinal and micro-longitudinal studies suggest a stronger and more consistent unidirectional effect of sleep on pain exacerbation of pain in adult patients, particularly in experimental and acute pain models (28–32). For example, it has been shown that sleep deprivation can elicit hyperalgesic responses (i.e., abnormally increased pain sensitivity) in healthy individuals that correlate with electrophysiologic measures (e.g., decreases in laser-evoked potentials) (33) and that some of these responses can be reversed by a nap or short sleep regardless of vigilance status (34).

The relationship between the central and peripheral modulation of pain and the ANS is becoming clearer as its mechanisms of action deepen. Although the main function of the ANS is not to directly regulate pain, it can influence the experience of pain and alter the perception of pain by activating the sympathetic nervous system during stress (35, 36). For example, acute pain increases an autonomic response such as respiratory rate, leads to muscle tension, increases electrodermal activity and dilates the pupils (37). Similarly, activation of the parasympathetic nervous system via the vagus nerve and the inflammatory reflex can reduce pain levels (38–40). At the central level, pain experiences link the low frequencies of heart rate variability to the brain connectivity of some important areas in the perception and modulation of pain, such as the PAG and the dorsal anterior cingulate cortex (41).

The vagus nerve is a mixed nerve consisting of sensory, motor and parasympathetic fibers and contains approximately 80% afferent fibers and 20% efferent fibers (42). Of the three types of fibers belonging to the vagus nerve, namely the A fibers (myelinated) and the B fibers (preganglionic innervation), the unmyelinated C fibers (which make up about 70% of all vagus fibers) are responsible for the transmission of visceral information of various visceral organs, which have shown a modulating effect on the pain produced by the vagus nerve (43, 44). Due to the importance of the vagus nerve in the modulation of pain, various approaches to the treatment of chronic pain have been considered, which can be divided into invasive and non-invasive methods. The latter have prevailed because they are more convenient to use and involve fewer risks in handling and consequently fewer clinical complications (10, 45).

Although the vagus nerve is an extremely important nerve, it is just another part of a truly complex and global system. Therefore, there is an urgent need to develop solutions that allow neuromodulation of the ANS to restore its normal function and thus its pain modulation.

The modern era of neuromodulation began with the publication of a study by Benabid et al. in 1987, in which deep brain stimulation was used to suppress tremor in Parkinson's disease (46). However, the use of electricity to treat neurological disorders dates back to the earliest antiquity (47). As early as the 1st century BC, the physician Scribonius Largus recommended shocks with the torpedo fish marmorata to the Emperor Claudius to treat his headaches and ailments (48). Advances in neuroimaging with the advent of magnetic resonance imaging and functional imaging, together with improved surgical techniques, have contributed to a significant development of these techniques and greater precision in their application (49). A better understanding of the neural circuitry involved in various neurological, psychiatric, cognitive and behavioral disorders has led to an expansion of the application of neuromodulation techniques to a considerable number of nervous system disorders (50, 51).

Especially for pain, neuromodulation can be divided into invasive, minimally invasive and non-invasive methods, with the focus on electrical stimulation. the three types of neuromodulation are effective in the treatment of pain, but due to the potential risks and complications of invasive or minimally invasive procedures, non-invasive interventions have been presented as the first choice for pain management (52, 53). Until a few years ago, two types of non-invasive neuromodulation were known and researched: peripheral and central (54–57) in which different types of electrical generators were used and are still being investigated. The use of microcurrents as a non-invasive neuromodulation in the treatment of pain has proven to be increasingly successful and is being used more and more frequently as a therapeutic tool in different centers specializing in acute or chronic pain (58–60). The vast majority of microcurrent devices are applied locally, which is a limitation in polyarticular or systemic conditions. Nowadays, the most studied and traditional (non-invasive) neuromodulation technologies for pain are direct tDCS, which is a central neuromodulation, and peripheral vagal neuromodulation (9, 10). Although both use electrical microcurrents and are applied transcutaneously, neither focuses specifically on autonomic modulation. On the other hand, they cover only one input target, central or peripheral. NESA microcurrents go one step further. It is a neuromodulation that has a global capacity (central and peripheral). This means that modulatory actions such as orthodromic impulses that activate descending inhibitory pathways, afferent and efferent regulatory mechanisms of neuromodulatory systems and neurotransmitters, and finally the modulatory capacity of the ANS can be generated systemically using the control component (56, 61, 62).

Non-invasive neuromodulation with NESA is a superficial treatment that provides a highly comfortable experience for the patient (50). The effect of the electrical microcurrent is amplified as it is delivered through multiple pathways covering the entire body via limb electrodes and a directing electrode. The NESA technique uses 24 coordinated electrodes to modulate the autonomic nervous system (ANS) through ultra-low-frequency electrical signals.

These signals range from 1.4 Hz to 14.28 Hz, depending on the program, with pulse intensities between 0.1 and 0.9 milliamperes and a potential difference of ±3 V. The current is distributed in sequences (100–130 ms per program) with biphasic and monophasic alternations, activating different polarities over the course of the session (12).

**Figure 1:**
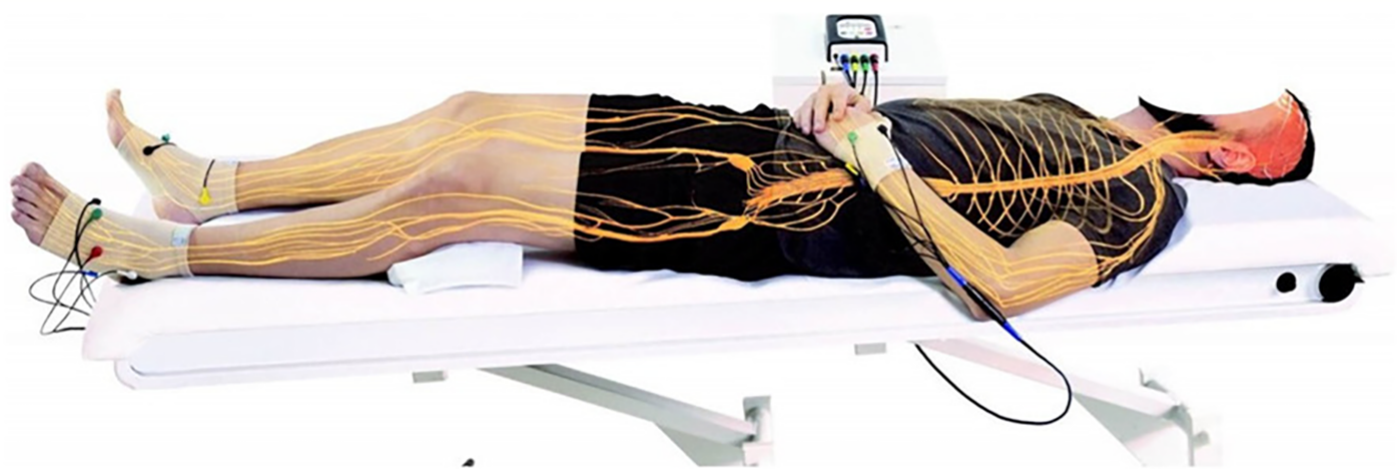
Graphical representation of the position of the NESA microcurrent electrodes.

Unlike local muscle or nerve stimulation, NESA microcurrents produce a systemic effect due to the distributed electrode placement and the extremely low electrical parameters. The technique utilizes 24 semi-electrodes—six per limb, with specialized gloves for the wrists and anklets for the ankles—delivering synchronized stimulation. This coordinated electrical input effectively modulates the ANS while remaining entirely non-invasive, as it is applied only to the body's surface (12) (see Figure 1).

## 3 Discussion

Based on the evidence and clinical experience to date, non-invasive neuromodulation NESA can also be a complementary treatment to other therapies. Two types of treatment modalities can be described: passive and active treatment. Passive treatment modulates the autonomic nervous system, while the application of our therapies improves the treatment. It is also a way to familiarize the patient with the technology and see what the potential is. In active treatment, doctors can combine different therapies with the electrical modulation of the ANS through NESA non-invasive neuromodulation. Treatment with NESA microcurrents begins with the patient's medical history. The collection of all information enables the definition of short, medium and long-term goals. The programming of the device focuses on the first target, and once this first target is achieved, it is programmed to treat the second target. As a general parameter, a low voltage application is recommended, based on the latest studies (63–65).

The treatment times are set at one hour. The recommended frequency of application is dependent on the profesionaĺs clinical reasoning and the patient's necessity. Exist treatment protocols with twice (64), three (63, 65) during the whole treatment, in addition, what have been demonstrated is that at least in necessary 10 sessions (63–65). NESA treatment is considered as a middle-long treatment in order to modulate the ANS. If the course of treatment is favorable, the sessions can be carried out at longer intervals or more frequently, always depending on the clinical reasoning (12).

The technology may be a useful and effective tool in high-level players to optimize recovery and content with exercise stressors (64). In addition, non-invasive neuromodulation has been shown to be a safe technique for children, with any adverse effect registered, that is easy to apply and shows clinically relevant results in autonomic systems such as sleep and constipation (63) or cognitive function and sleepiness in dementia patients (65) as well as promising results in chronic diseases. In line with these findings, studies in recent years have explored new possibilities for the treatment of pain and sleep quality in patients with chronic pain, such as multiple sclerosis (8), with positive results in pain reduction and sleep quality. In all published studies the authors describe that no adverse effects are recorded, this is probably due to the characteristics of microcurrents which are usually a very patient-friendly treatment. Based on these results, some clinical trials are currently being conducted in others diseases such as fybromialgia sindrome (clinical trial registration NCT05648695), stroke sequeales (clinical trial registration NCT058539529), in patients with long covid (clinical trial registration NCT056814559), in pain after surgery (clinical trial registration NCT05207943) or even in complex regional syndrome (Sudeck) (clinical trial registration NCT05052736).

Although future research needs to be conducted in a variety of settings and patients, with larger sample sizes, and even in studies comparing groups with different neuromodulation techniques and NESA technology. It could demonstrate a way to treat symptoms such as pain from the perspective of targeting the patient's autonomic nervous system. There is still work to do but a very interesting line of research and clinical practice has been initiated based on a new view of treating pain from the perspective of targeting the patient's autonomic nervous system. Perhaps NESA microcurrents can help us to change the approach to pain management.
